# Efficacy and safety of risankizumab in patients with moderately to severely active Crohn’s disease: interim results from the SEQUENCE open-label extension study

**DOI:** 10.1093/ecco-jcc/jjaf213

**Published:** 2025-12-04

**Authors:** Laurent Peyrin-Biroulet, Raja Atreya, Silvio Danese, James O Lindsay, J Casey Chapman, Toni Anschutz, Xiu Huang, Javier Zambrano, Stijn van Haaren, Namita Joshi, W Rachel Duan, Raymond K Cross

**Affiliations:** Department of Gastroenterology, CHRU Nancy, INSERM NGERE, Université de Lorraine, F-54500 Vandœuvre-lès-Nancy, France; Division of Gastroenterology and Hepatology, McGill University Health Centre, Montreal, Quebec, Canada; Department of Medicine 1, Friedrich-Alexander-Universität Erlangen-Nürnberg, Erlangen, Germany; Gastroenterology and Endoscopy, IRCCS Ospedale San Raffaele and University Vita-Salute San Raffaele, Milan, Italy; Centre for Immunobiology, Barts and the London School of Medicine and Dentistry, Queen Mary University of London, London, United Kingdom; Crohn’s and Colitis Center at the Baton Rouge General and the GI Alliance, Baton Rouge, LA, United States; AbbVie Inc., North Chicago, IL, United States; AbbVie Inc., North Chicago, IL, United States; AbbVie Inc., North Chicago, IL, United States; AbbVie Inc., North Chicago, IL, United States; AbbVie Inc., North Chicago, IL, United States; AbbVie Inc., North Chicago, IL, United States; Melissa L. Posner Institute for Digestive Health & Liver Disease at Mercy Medical Center, Baltimore, MD, United States

**Keywords:** clinical trials, quality of life, biomarkers

## Abstract

**Background and Aims:**

Risankizumab, a selective interleukin-23 p19 inhibitor, is approved to treat moderately to severely active Crohn’s disease (CD) in adults. We report interim results from part 2 of the ongoing SEQUENCE trial evaluating long-term efficacy and safety of risankizumab in patients with active CD and previous anti–tumor necrosis factor failure.

**Methods:**

Patients randomized to risankizumab who completed the part 1 Week 48 visit could continue receiving open-label subcutaneous risankizumab 360 mg every 8 weeks (part 2). Patients with inadequate response could receive rescue therapy (intravenous risankizumab 600 mg) before continuing regular treatment. This interim analysis assessed efficacy at Weeks 52, 76, and 100 of treatment; safety was evaluated throughout.

**Results:**

Overall, 224 patients who received risankizumab 600 mg intravenous induction therapy and 360 mg subcutaneous maintenance therapy entered part 2. Clinical remission rates remained stable through Week 100 (as observed, ≥74.5%; nonresponder and modified non–responder imputation analyses showed similar trends). Most patients (>99%) achieving clinical remission were corticosteroid-free at the corresponding visit. CD-related hospitalization and surgery incidence were low (≤0.03 *n*/patient year), and Inflammatory Bowel Disease Questionnaire and 36-Item Short Form Health Survey improvements were sustained. Safety data were consistent with the known risankizumab safety profile; the exposure-adjusted serious adverse event rate was 11.8/100 patient-years.

**Conclusions:**

This interim analysis of continuous open-label risankizumab therapy showed durable long-term clinical efficacy and no new safety signals in patients with moderately to severely active CD. Future analyses will evaluate longer-term clinical and endoscopic outcomes and safety.

**Clinical trial registration number:**

NCT04524611

## 1. Introduction

Crohn’s disease (CD) is a progressive and destructive inflammatory bowel disease.^1^ The incidence of CD has increased globally since the beginning of the 21st century.^1^ The goal of CD treatment is to disrupt the progressive nature of the disease that may lead to intestinal failure and complications, to achieve sustained clinical and endoscopic remission and normalize quality of life.^2^ The management of CD has changed over time with newly available treatments.^3^ Advanced therapies have provided an effective option for treating CD, and ongoing studies are conducted on their safety and efficacy for long-term use.^4^ Anti–tumor necrosis factor (TNF) antibodies are often used as a first-line treatment for moderately to severely active CD. However, patients who demonstrate primary or secondary nonresponse, or who experience intolerance to their initial anti–TNF therapy, may benefit from an advanced therapy with a different mechanism of action.^5–7^

Risankizumab is a humanized IgG1 monoclonal antibody that specifically inhibits interleukin (IL)-23 by binding to its p19 subunit. It was designed to have low immunogenicity and high bioavailability, thus allowing for less frequent dosing; clinical implications of these modifications have not been studied.^8^^,^^9^ Risankizumab was well tolerated and efficacious for the treatment of moderately to severely active CD in the phase 3 ADVANCE and MOTIVATE trials and the 52-week maintenance FORTIFY phase 3 trial.^10^^,^^11^ In part 1 of the head-to-head SEQUENCE trial comparing the efficacy of risankizumab to ustekinumab, risankizumab demonstrated noninferiority to ustekinumab in inducing clinical remission at Week 24 and superiority over ustekinumab in achieving endoscopic remission at Week 48 among patients with CD and prior anti–TNF failure.^7^

As no curative treatments for CD are available, most patients require long-term treatment.^2^ Long-term treatment targets include clinical remission, endoscopic healing, the absence of disability, and improvements in quality of life, with patient-reported outcomes as a key measurement.^12^ Part 2 of the SEQUENCE study, the open-label extension (OLE) study, is investigating the long-term efficacy and safety of continued risankizumab treatment in patients with moderately to severely active CD who have had an inadequate or intolerant response to anti–TNF therapy. Herein, we present interim results from the first year of the ongoing SEQUENCE OLE study (through 100 weeks of risankizumab treatment).

## 2. Methods

### Study design, patients, and treatment

**Figure 1.**
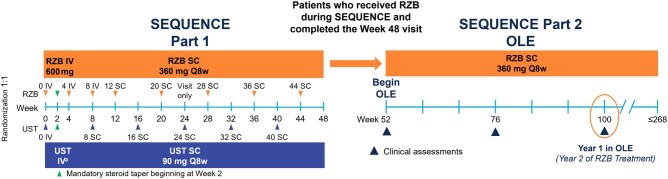
Study design. The study drug was administered open label. Stratification factors were number of prior failed anti–TNF agents (1, >1) and corticosteroid use at baseline (yes, no). ^a^UST baseline IV dose was weight-based: ≤55 kg, 260 mg; >55-85 kg, 390 mg; or >85 kg, 520 mg. IV, intravenous; OLE, open-label extension; Q8w, every 8 weeks; RZB, risankizumab; SC, subcutaneous; UST, ustekinumab.

SEQUENCE (NCT04524611) is an ongoing phase 3b, multicenter, open-label, efficacy assessor–blinded, randomized trial in which patients with moderately to severely active CD and an inadequate response or intolerance to anti–TNF therapy were randomly assigned to receive risankizumab or ustekinumab for 48 weeks (part 1); trial details, including eligibility criteria, were previously reported.^7^ Part 2 began at the first dose administered, typically scheduled for Week 52 (Figure 1). Patients who were randomized to the risankizumab arm in part 1 and completed the Week 48 visit could continue receiving open-label subcutaneous (SC) risankizumab 360 mg maintenance doses every 8 weeks in part 2 (the OLE study) of the SEQUENCE trial; patients randomized to the ustekinumab arm in part 1 were not eligible for part 2 and were discontinued from the study. At the Week 52 visit, patients received risankizumab syringes for at-home injections every 8 weeks (as allowed per local requirements).

Patients with inadequate response during part 2 could receive rescue therapy (1 dose of intravenous [IV] risankizumab 600 mg, then SC risankizumab 360 mg every 8 weeks). Inadequate response was defined as average daily stool frequency (SF) ≥3.3 and/or average daily abdominal pain (AP) score ≥1.5 plus an objective marker of inflammation. Objective markers of inflammation included high-sensitivity C-reactive protein (hs-CRP) concentration of ≥5 mg/L, fecal calprotectin (FCP) concentration of ≥250 μg/g, or a simple endoscopic score for Crohn’s disease (SES-CD), excluding the narrowing component, of ≥6 (≥4 for isolated ileal disease). Patients could receive rescue therapy at any point during part 2 and could receive up to 2 rescue visits per year ≥16 weeks apart.

This study was conducted in accordance with the Good Clinical Practice Guideline as defined by the International Conference on Harmonisation, The Declaration of Helsinki, and all applicable federal and local regulations. An independent ethics committee or institutional review board approved the protocol at each trial site. All patients provided written informed consent.

### Assessments

#### Efficacy assessments

Efficacy outcomes were evaluated at Weeks 52, 76, and 100 from the baseline visit of SEQUENCE part 1. The Week 52 efficacy results were from the most recent non–missing assessment conducted before the initial risankizumab dose in part 2. Clinical outcomes included achievement of clinical remission per Crohn’s Disease Activity Index (CDAI; defined as CDAI < 150), and achievement of clinical remission per SF/APS (defined as an average daily SF score of ≤2.8 and an average daily AP score of ≤1 [with both not worse than baseline of part 1]). The achievement of corticosteroid-free clinical remission, defined as the achievement of clinical remission without corticosteroids on the day of the corresponding visit, was assessed as a prespecified endpoint; 90-day corticosteroid-free clinical remission, defined as the achievement of clinical remission with no corticosteroids received within 90 days before the corresponding visit, was analyzed post hoc (not prespecified). Biomarker normalization was assessed post hoc (not prespecified) and defined as achieving hs-CRP levels ≤5 mg/L or FCP levels ≤250 μg/g among patients with elevated biomarkers at baseline. The incidence rate of CD-related hospitalizations and surgeries in part 2 was also assessed. In part 2, endoscopies are performed at Week 148 and at the end of part 2, and, therefore, are not included in this Week 100 interim analysis.

Health-related quality-of-life outcomes included achieving Inflammatory Bowel Disease Questionnaire (IBDQ) response (defined as an increase in total score of ≥16 points from baseline), achieving IBDQ remission (defined as a total score ≥170 points), achieving a clinically meaningful improvement from baseline on the 36-Item Short Form Health Survey (SF-36) physical component score (PCS; defined as an increase of ≥4.1 points from baseline), and achieving a clinically meaningful improvement from baseline on the SF-36 mental component score (MCS; defined as an increase of ≥3.9 points from baseline).

#### Safety assessments

Safety evaluations included monitoring for adverse events (AEs), conducting physical examinations, measuring vital signs, and performing clinical laboratory testing (hematology and chemistry measurements) throughout the study. An independent, blinded, expert external committee adjudicated cardiovascular events and suspected anaphylactic events based on prespecified definitions.

### Statistical analysis

This interim analysis presents data through Week 100 of risankizumab treatment (data cutoff date: July 11, 2024). Data were assessed for all patients in the part 2 intent-to-treat population (patients who received the risankizumab 600 mg IV followed by risankizumab 360 mg SC regimen in part 1 and ≥1 dose of risankizumab in part 2). Efficacy outcomes were evaluated using as-observed (AO), non–responder imputation (NRI), and modified NRI (mNRI) analyses. AO analyses are reported in the text; AO, NRI, and mNRI analyses are shown in figures. For AO analyses, missing evaluations were not imputed, and only evaluations before the start of rescue therapy were included. AO analyses for the rescue therapy group included data from the date of rescue; visits were recalibrated to every 24 weeks from the date of the initial rescue therapy. For NRI analyses, patients were categorized as nonresponders for visits with missing assessments, visits after premature discontinuation of the study, and visits after initiation of rescue therapy (if applicable). For mNRI analyses, missing data was addressed using multiple imputation for missing assessments (including missing values following premature discontinuation of study treatment for reasons other than lack of efficacy or an AE limited to CD); patients were categorized as nonresponders for visits after premature discontinuation from treatment due to lack of efficacy or an AE limited to CD, and for visits on or after the initiation of rescue therapy. Safety analyses included all patients who received ≥1 dose of risankizumab in the study. No statistical comparisons were conducted; all analyses, including safety, were summarized with descriptive statistics.

## 3. Results

### Patients

A total of 224 patients who received risankizumab 600 mg IV followed by risankizumab 360 mg SC regimen in part 1 entered part 2 of the SEQUENCE trial. Of these, 26 (11.6%) patients discontinued treatment before the data cutoff date, including 6 (2.7%) due to AEs, 6 (2.7%) due to lack of efficacy, 7 (3.1%) due to patient withdrawal, 6 (2.7%) due to other reasons, and 1 (0.4%) who was lost to follow-up. The demographics of the patient population in part 2 were similar to those of the patient population in part 1. The majority of patients were male (53.1%) and White (77.0%) (Table 1). The median (interquartile range) length of CD duration was 8.6 (10.5) years, 23.7% of patients had >1 anti–TNF failure, and 21.4% of patients were using corticosteroids at baseline. The relatively low CDAI and SES-CD values at the beginning of part 2 are consistent with over half of patients achieving clinical remission and nearly a third of patients achieving endoscopic remission in part 1 of the SEQUENCE trial (Week 48).^7^

**Table 1.** Patient demographics and disease characteristics at Week 52 (beginning of part 2)^a^.

Characteristic	Prior treatment with IV 600 mg/SC 360 mg RZB (*N* = 224)
**Age, years, mean (SD)**	38.3 (13.2)
**Female, *n* (%)**	105 (46.9)
**Male, *n* (%)**	119 (53.1)
**Race, *n* (%)**	
** White**	171 (77.0)
** Asian**	43 (19.4)
** Black or African American**	6 (2.7)
** Other^b^**	4 (1.8)
**BMI, kg/m^2^, mean (SD)**	24.9 (5.2)
**CD duration, years, median (range)**	8.6 (1.5-41.6)
**CD location, *n* (%)**	
** Ileal**	32 (14.3)
** Colonic**	92 (41.1)
** Ileocolonic**	100 (44.6)
**FCP, μg/g, median (IQR)**	152.5 (475.0)
**hs-CRP, mg/L, median (IQR)**	3.1 (5.4)
**Average daily SF, mean (SD)**	1.5 (1.9)
**Average daily APS, mean (SD)**	0.5 (0.6)
**CDAI, mean (SD)**	102.4 (83.2)
**SES-CD, mean (SD)**	5.8 (5.8)
**IBDQ total score, mean (SD)^c^**	175.6 (30.4)
**SF-36 PCS, mean (SD)**	50.0 (7.5)
**SF-36 MCS, mean (SD)**	47.4 (9.3)
**Prior anti**–**TNF failure at baseline of part 1, *n* (%)**	
** 1**	171 (76.3)
** >1**	53 (23.7)
**Corticosteroid use at baseline of part 1, *n* (%)**	48 (21.4)
**Immunomodulator use at baseline of part 1, *n* (%)**	33 (14.7)

Abbreviations: APS, abdominal pain score; BMI, body mass index; CD, Crohn’s disease; CDAI, Crohn’s Disease Activity Index; FCP, fecal calprotectin; hs-CRP, high-sensitivity C-reactive protein; IBDQ, Inflammatory Bowel Disease Questionnaire; IQR, interquartile range; IV, intravenous; MCS, mental component summary; PCS, physical component summary; RZB, risankizumab; SC, subcutaneous; SES-CD, Simple Endoscopic Score for Crohn’s Disease; SF, stool frequency; SF-36, 36-Item Short Form Health Survey; TNF, tumor necrosis factor.

a Unless otherwise indicated.

b Includes American Indian/Alaskan Native, Native Hawaiian or other Pacific Islander, multiple, or missing.

c Patient-reported outcome.

As of the data cutoff date, 16 (7.1%) patients had received rescue therapy during part 2, and 4 (25.0%) of these patients had discontinued treatment. Demographics and characteristics of patients who received rescue therapy are shown in Supplementary Table S1.

### Efficacy assessments

**Figure 2.**
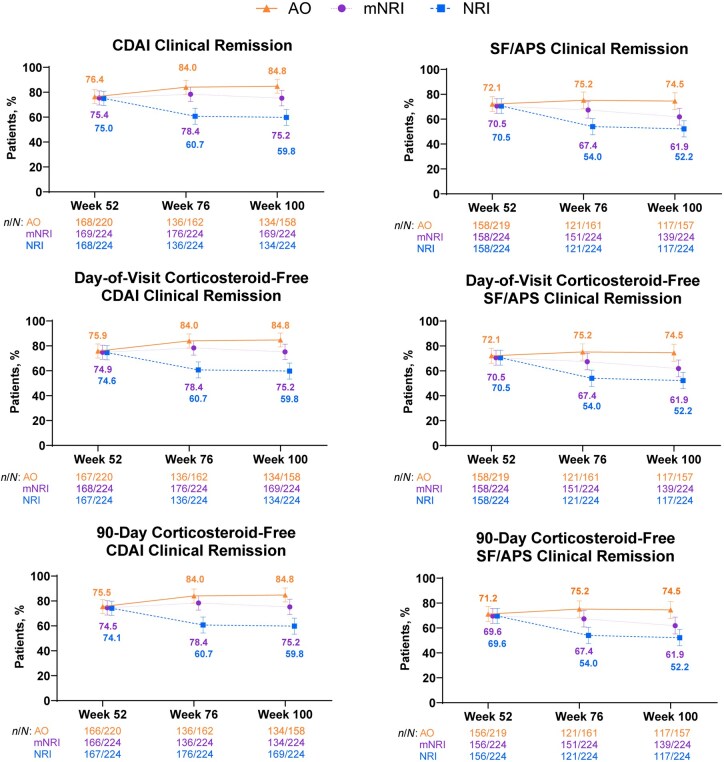
Clinical remission and corticosteroid-free clinical remission. Error bars are 95% CI. CDAI clinical remission was defined as CDAI <150. SF/APS clinical remission was defined as average daily SF ≤2.8 and not worse than baseline of the induction study, and average daily APS ≤1 and not worse than baseline of the induction study. Day-of-visit corticosteroid-free was defined as no corticosteroids received at the corresponding visit. A 90-day corticosteroid-free period was defined as no corticosteroids received within 90 days before the scheduled visit. AO, as observed; APS, abdominal pain score; CDAI, Crohn’s Disease Activity Index; mNRI, modified non–responder imputation; NRI, non–responder imputation; SF, stool frequency.

Clinical remission rates remained stable during part 2 (Figure 2). CDAI clinical remission rates were ≥76%, and SF/APS clinical remission rates were ≥72% from Week 52 through Week 100. In a post hoc analysis by disease location, clinical remission rates were highest among patients with colonic-only disease relative to those with ileal-only or ileocolonic disease (Supplementary Table S2); a smaller number of patients had ileal-only disease. Most patients who received rescue therapy were able to achieve clinical remission (Supplementary Table S3); CDAI and SF/APS clinical remission rates in patients who received rescue therapy were ≥57% at 24 and 48 weeks after their initial rescue therapy. Most patients (≥99%) who achieved clinical remission were not receiving corticosteroids at the corresponding visits or 90 days before each scheduled visit (Figure 2). Patients who achieved clinical remission at the beginning of part 2 demonstrated sustained clinical remission, with 92.1% of those who achieved CDAI clinical remission at Week 52 achieving CDAI clinical remission at Week 100 and 86.3% of those who achieved SF/APS clinical remission at Week 52 achieving SF/APS clinical remission at Week 100 (Supplementary Figure S1).

**Figure 3.**
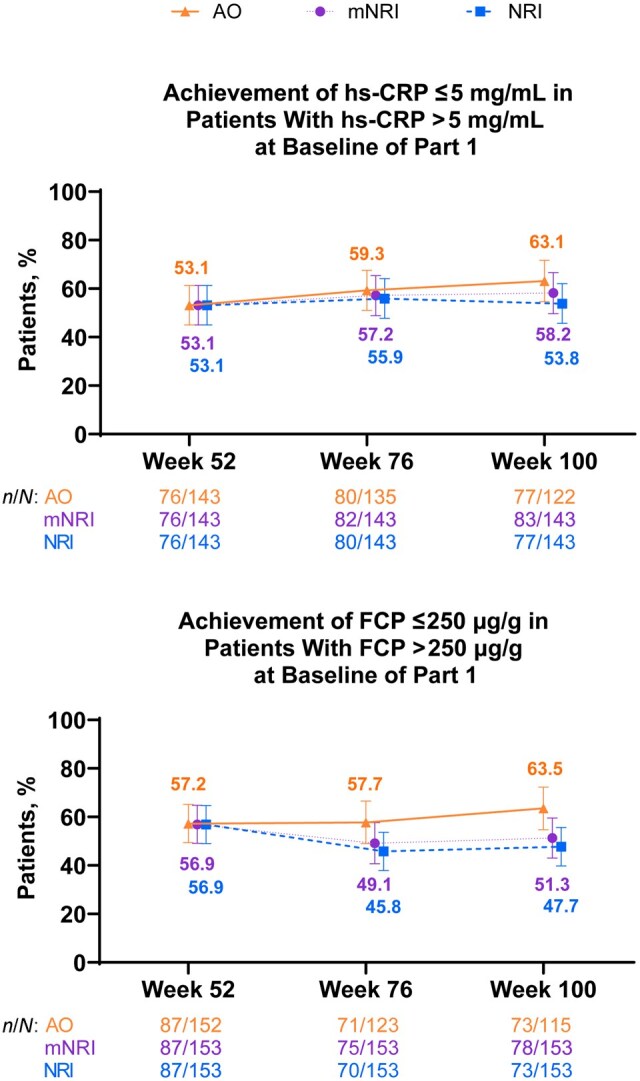
Biomarker normalization. Error bars are 95% CI. AO, as observed; FCP, fecal calprotectin; hs-CRP, high-sensitivity C-reactive protein; mNRI, modified non–responder imputation; NRI, non–responder imputation.

The majority of patients with elevated biomarkers at baseline achieved normalization of hs-CRP (63.1%) or FCP levels at Week 100 (63.5%) (Figure 3); absolute hs-CRP and FCP concentrations and changes from baseline are shown in Supplementary Table S4. The exposure-adjusted incidence rate of CD-related hospitalizations through the data cutoff was 0.03 *n*/patient-years (PY) (95% confidence interval [CI], 0.01, 0.05). The exposure-adjusted incidence rate of CD-related surgeries through the data cutoff was 0.01 *n*/PY (95% CI, −0.001, 0.02).

**Figure 4.**
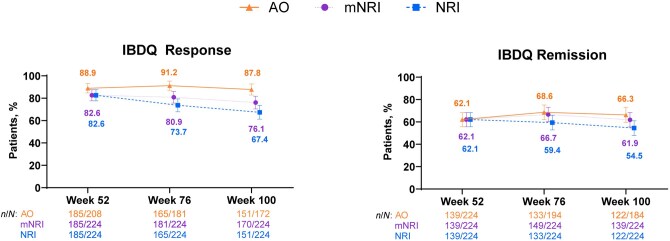
IBDQ Response and remission. Error bars are 95% CI. IBDQ response was defined as an increase in IBDQ total score of ≥16 points from the baseline of part 1. IBDQ remission was defined as an IBDQ total score ≥170 points. AO, as observed; IBDQ, Inflammatory Bowel Disease Questionnaire; mNRI, modified non–responder imputation; NRI, non–responder imputation.

**Figure 5.**
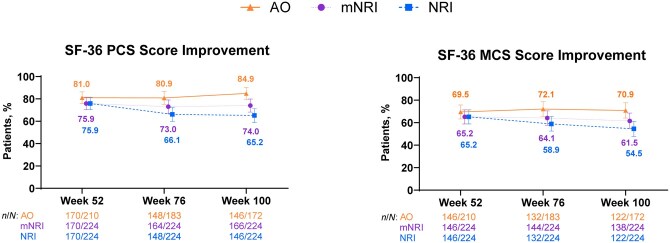
SF-36 PCS and MCS score improvement. Error bars are 95% CI. SF-36 PCS improvement was defined as an increase of ≥4.1 points from baseline of part 1. SF-36 MCS improvement was defined as an increase of ≥3.9 points from baseline of part 1. AO, as observed; MCS, mental component summary; mNRI, modified non–responder imputation; NRI, non–responder imputation; PCS, physical component summary; SF-36, 36-Item Short Form Health Survey.

Patients demonstrated sustained and clinically meaningful improvements in IBDQ through Week 100, as assessed by IBDQ response (≥87%) and IBDQ remission (≥62%) (Figure 4). Likewise, patients demonstrated sustained and clinically meaningful improvements in SF-36 PCS (≥80%) and MCS (≥69%) through 100 weeks of risankizumab treatment (Figure 5). Similar trends were observed with mNRI and NRI analyses for all efficacy outcomes.

### Safety

The most common treatment-emergent adverse events (TEAEs) per 100 patient-years (E/100 PY) were COVID-19 (17.2), worsening of CD (7.3), and nasopharyngitis (7.6). The exposure-adjusted event rate (E/100PY; Table 2) was 11.8 for serious AEs, 2.7 for AEs leading to the discontinuation of study drug, 2.9 for serious infections, and 0.7 for malignant tumors. No deaths were reported during the study. No adjudicated major adverse cardiovascular events, active tuberculosis, or serious hypersensitivity events were reported. These data confirm the safety profile previously described with risankizumab treatment.

**Table 2.** Treatment-emergent adverse events during the SEQUENCE trial (parts 1 and 2).

Treatment-emergent adverse events	RZB (*N* = 262, PYs = 591.4) E (E/100 PY)
**Overall TEAE**	
** Any TEAE**	1386 (234.3)
**TEAE related to study drug according to the investigator**	235 (39.7)
** Severe TEAE**	109 (18.4)
** Serious TEAE**	70 (11.8)
** TEAE leading to discontinuation of study drug**	16 (2.7)
** Death**	0
**TEAE of special interest**	
** Adjudicated MACE events**	0
** Active tuberculosis**	0
** Serious infections**	17 (2.9)
**Opportunistic infections excluding tuberculosis and herpes zoster**	2 (0.3)
** Malignant tumors**	4 (0.7)^a^
** Herpes zoster**	3 (0.5)^b^
** NMSC**	2 (0.3)
** Hypersensitivity**	52 (8.8)^c^
** Serious hypersensitivity**	0
** Adjudicated anaphylactic reaction**	0
** Hepatic events**	36 (6.1)^c^ ^,d^
** Injection-site reactions**	13 (2.2)^c^

Safety summary includes all patients who received at least 1 dose of the study drug. TEAEs are defined as events that begin either on or after the first dose of the study drug within part 1 or 2 of SEQUENCE trial and within 140 days after the last dose of study drug in part 2 for patients who did not participate in CTE or until the first dose of the CTE if the patient was enrolled into CTE.

Abbreviations: CTE, continuous trial extension; E, events; MACE, major adverse cardiovascular event; NMSC, nonmelanoma skin cancer; PY, patient-years; RZB, risankizumab; TEAE, treatment-emergent adverse event.

a Four malignancy events were reported: plasma cell myeloma, basal cell carcinoma, squamous cell carcinoma, and breast cancer, each in 1 patient.

b All 3 herpes zoster events were mild or moderate and did not lead to study drug discontinuation.

c No events of hypersensitivity, hepatic events, or injection-site reactions led to study drug discontinuation.

d No events met Hy’s law criteria.

In the group that received rescue therapy, the TEAE profile was generally consistent with the all-risankizumab population. The exposure-adjusted event rate was 27.6 for serious AEs, 0 for AEs leading to the discontinuation of study drug, and 6.9 for serious infections. No malignant tumors, adjudicated major adverse cardiovascular events, active tuberculosis, or serious hypersensitivity events were reported in this group. No new safety risks were identified in this population.

## 4. Discussion

Patients with moderately to severely active CD who received 2 years of continuous risankizumab therapy in the SEQUENCE trial demonstrated durable clinical efficacy and quality-of-life benefits, without new safety signals. Clinical remission per CDAI and per SF/APS were sustained through 100 weeks. These results are consistent with those from the phase 3 FORTIFY OLE study evaluating the long-term safety and efficacy of risankizumab in over 1100 patients with moderately to severely active CD, in which AO and NRI clinical remission rates were maintained with risankizumab from Weeks 56 to 152 of treatment.^13^

No new safety risks were identified in this interim analysis of part 2 of the SEQUENCE trial evaluating the safety and efficacy of risankizumab in patients with moderately to severely active CD. The rates of AEs of special interest were generally comparable to the results of the FORTIFY OLE study (up to 104 and 152 weeks) of risankizumab treatment in patients with moderately to severely active CD.^13^^,^^14^ Serious infection and malignancy rates were consistent with previously published estimates for comparable patient populations. For example, the rate of serious infection observed in this OLE study (2.9 E/100 PY) is consistent with the incidence rate of serious infections (2.98 per 100 PY in CD) reported in a register-based cohort study.^15^ The observed rate of malignancies in risankizumab-treated patients with CD in this study (0.7 E/100 PY for malignant tumors) is within the expected range (0.98 per 100 PY for cancer excluding NMSC) for this patient population based on published estimates.^16^ Overall, the observed safety profile is consistent with the known risankizumab safety profile in CD, ulcerative colitis, psoriasis, and psoriatic arthritis, and supports long-term risankizumab treatment.^13^^,^^17–20^

Nearly all patients who achieved CDAI and SF/APS clinical remission met the corticosteroid-free criteria, regardless of whether corticosteroid use was assessed during the same visit or over the preceding 90 days, demonstrating the potential for effective disease control without corticosteroids. These results further support those from part 1 of the SEQUENCE trial, in which all patients who achieved CDAI clinical remission also achieved corticosteroid-free CDAI clinical remission at Week 48.^7^ The majority of patients in the current study demonstrated biomarker normalization, which is consistent with observations from previous clinical studies of risankizumab and offers an additional insight into therapeutic response despite the lack of available endoscopic data for this analysis.^21^ CD-related hospitalizations and surgeries in part 2 of the SEQUENCE trial were rare; the incidence rate for hospitalizations was similar to that reported for part 1 (the incidence rate for surgeries was not reported for part 1).^7^

Patients in part 2 demonstrated IBDQ response and remission, as well as clinically meaningful improvements from baseline in SF-36 scores, indicating sustained health-related quality-of-life benefits over time. These data were consistent with those in the FORTIFY OLE study, in which patients who received SC risankizumab sustained improvements in IBDQ total score relative to baseline of induction through 104 weeks.^14^

Patients who received rescue therapy also achieved clinical remission per CDAI and SF/APS. Despite the small sample size due to the limited number of patients needing rescue, these results indicate that patients who receive rescue therapy can achieve clinical remission after loss of response. Dose escalation (eg, to more frequent dosing) is a common treatment strategy to maintain or regain response for patients who experience a loss of response.^22^ Although it may be effective, dose escalation can lead to higher costs for the patient and the healthcare system.^23^ As IV reinduction may be a more viable real-world strategy than dose escalation, the observed efficacy after rescue therapy in this study may support IV reinduction as a potential option for patients with loss of response or incomplete response.

All open-label designs limit interpretations because of the risk of bias. As patients who were treated with ustekinumab in part 1 were not eligible for part 2, these results lack a control or comparator group, which limits conclusions about the relative efficacy or safety of risankizumab therapy in this population compared with other treatments in the longer term. Missing data are also common in long-term extension studies. To help mitigate these limitations, AO, NRI, and mNRI results are reported. Although the small number of patients receiving rescue therapy limits conclusions regarding those patients, the small number of patients requiring rescue therapy indicates the clinical benefit of dosing SC risankizumab 360 mg every 8 weeks for most patients. Endoscopic data were unavailable for this interim data analysis, as endoscopies are performed at Week 148 and at the completion of part 2; the lack of endoscopic data limits the interpretability of these results in the context of STRIDE-II long-term treatment targets.^12^ As the OLE study continues, endoscopy data will be collected along with efficacy endpoints over longer time periods. Lastly, the sponsor’s participation in the study design, data analysis, and writing of this report may introduce potential bias.

In this OLE study of the SEQUENCE trial, risankizumab demonstrated sustained clinical efficacy and quality-of-life benefits through 100 weeks of treatment among patients with moderately to severely active CD who had an inadequate response or experienced intolerance to anti–TNF therapy. The observed safety profile was consistent with the known risankizumab safety profile, and together, these findings further support long-term risankizumab treatment for CD.

## Supplementary Material

jjaf213_Supplementary_Data

## Data Availability

AbbVie is committed to responsible data sharing regarding the clinical trials we sponsor. This includes access to anonymized individual and trial-level data (analysis data sets), as well as other information (eg, protocols, clinical study reports, or analysis plans), as long as the trials are not part of an ongoing or planned regulatory submission. This includes requests for clinical trial data for unlicensed products and indications. These clinical trial data can be requested by any qualified researchers who engage in rigorous, independent scientific research and will be provided after the review and approval of a research proposal and statistical analysis plan (SAP) and execution of a data sharing agreement (DSA). Data requests can be submitted any time after approval in the United States and Europe and after acceptance of this manuscript for publication. The data will be accessible for 12 months, with possible extensions considered. For more information on the process or to submit a request, visit the following link: https://vivli.org/ourmember/abbvie/, then select “Home.”

## Conference presentation

Part of this work was presented in an oral presentation at the 20th Congress of the European Crohn’s and Colitis Organisation, Berlin, Germany in 2025 [Peyrin-Biroulet L. et al. *J Crohns Colitis.* 2025; 19[Suppl1]: 1405-7].
