# Supplementary material for: Efficacy and safety of risankizumab in patients with moderately to severely active Crohn’s disease: interim results from the SEQUENCE open-label extension study
Source: J Crohns Colitis. 2025 Dec 4;19(12):jjaf213. doi: 10.1093/ecco-jcc/jjaf213 (PMC12726915; doi:10.1093/ecco-jcc/jjaf213)

SUPPLEMENTARY INFORMATION

Efficacy and Safety of Risankizumab in Patients with Moderately to Severely Active Crohn's Disease: Interim Results from the SEQUENCE Open-Label Extension Study

Laurent Peyrin-Biroulet,[a,b] Raja Atreya,[c] Silvio Danese,[d] James O. Lindsay,[e] J. Casey Chapman,[f] Toni Anschutz,[g] Xiu Huang,[g] Javier Zambrano,[g] Stijn van Haaren,[g] Namita Joshi,[g] W. Rachel Duan,[g] Raymond K. Cross[h]

[a]Department of Gastroenterology, CHRU Nancy, INSERM NGERE, Université de Lorraine, F-54500 Vandœuvre-lès-Nancy, France; [b]Division of Gastroenterology and Hepatology, McGill University Health Centre, Montreal, Quebec, Canada; [c]Department of Medicine 1, Friedrich-Alexander-Universität Erlangen-Nürnberg, Erlangen, Germany; [d]Gastroenterology and Endoscopy, IRCCS Ospedale San Raffaele and University Vita-Salute San Raffaele, Milan, Italy; [e]Centre for Immunobiology, Barts and the London School of Medicine and Dentistry, Queen Mary University of London, London, UK; [f]Crohn's and Colitis Center at the Baton Rouge General and the GI Alliance, Baton Rouge, LA, USA; [g]AbbVie Inc., North Chicago, IL, USA; [h]Melissa L Posner Institute for Digestive Health & Liver Disease at Mercy Medical Center, Baltimore, MD, USA

Supplementary Table 1. Demographics and disease characteristics at week 52 (beginning of part 2)[a] for patients who received rescue therapy

Characteristic	Prior treatment with IV 600 mg/SC 360 mg RZB (*n* = 16)
Age, years, mean (SD)	34.0 (15.7)
Female, *n* (%)	5 (31.3)
Male, *n* (%)	11 (68.8)
Race, *n* (%)	
White	14 (87.5)
Asian	2 (12.5)
Black or African American	0
Other[b]	0
BMI, kg/m^2, mean (SD)	22.8 (4.4)
CD duration, years, median (range)	5.8 (1.5–41.6)
CD location, *n* (%)	
Ileal	2 (12.5)
Colonic	8 (50.0)
Ileocolonic	6 (37.5)
FCP, µg/g, median (IQR)	927.0 (1844.0)
hs-CRP, mg/L, median (IQR)	4.2 (6.3)
Average daily SF, mean (SD)	2.1 (3.3)
Average daily APS, mean (SD)	0.6 (0.9)
CDAI, mean (SD)	128.5 (128.5)
SES-CD, mean (SD)	10.3 (10.1)
IBDQ total score, mean (SD)[c]	171.6 (23.9)
SF-36 PCS, mean (SD)	48.3 (6.8)

SF-36 MCS, mean (SD)	48.3 (7.7)
Prior anti-TNF failure at baseline of part 1, *n* (%)	
1	14 (87.5)
>1	2 (12.5)
Corticosteroid use at baseline of part 1, *n* (%)	7 (43.8)
Immunomodulator use at baseline of part 1, *n* (%)	4 (25.0)

APS, abdominal pain score; BMI, body mass index; CD, Crohn's disease; CDAI, Crohn's Disease Activity Index; FCP, fecal calprotectin; hs-CRP, high-sensitivity C-reactive protein; IBDQ, Inflammatory Bowel Disease Questionnaire; IQR, interquartile range; MCS, mental component summary; PCS, physical component summary; RZB, risankizumab; SES-CD, Simple Endoscopic Score for Crohn's Disease; SF, stool frequency; SF-36, 36-Item Short Form Health Survey; TNF, tumor necrosis factor.

[a]Unless otherwise indicated.

[b]Includes American Indian/Alaskan Native, Native Hawaiian or other Pacific Islander, multiple, or missing.

[c]Patient-reported outcome.

Supplementary Table 2. Achievement of clinical remission by disease location

Endpoint	Disease location	AO Week 52	AO Week 76	AO Week 100	mNRI Week 52	mNRI Week 76	mNRI Week 100	NRI Week 52	NRI Week 76	NRI Week 100
CDAI remission, *n*/*N* (%)	Ileal	20/35 (57.1)	16/26 (61.5)	16/25 (64.0)	20/35 (57.1)	21/35 (61.0)	19/35 (53.5)	20/35 (57.1)	16/35 (45.7)	16/35 (45.7)
	Colonic	76/90 (84.4)	68/74 (91.9)	64/71 (90.1)	77/92 (83.5)	79/92 (85.6)	77/92 (83.8)	76/92 (82.6)	68/92 (73.9)	64/92 (69.6)
	Ileocolonic	72/95 (75.8)	52/62 (83.9)	54/62 (87.1)	72/97 (74.2)	75/97 (77.6)	73/97 (75.6)	72/97 (74.2)	52/97 (53.6)	54/97 (55.7)
SF/APS remission, *n*/*N* (%)	Ileal	16/35 (45.7)	14/26 (53.8)	15/25 (60.0)	16/35 (45.7)	18/35 (51.7)	16/35 (46.4)	16/35 (45.7)	14/35 (40.0)	15/35 (42.9)
	Colonic	75/89 (84.3)	61/73 (83.6)	56/70 (80.0)	75/92 (81.5)	70/92 (75.9)	66/92 (71.5)	75/92 (81.5)	61/92 (66.3)	56/92 (60.9)
	Ileocolonic	67/95 (70.5)	46/62 (74.2)	46/62 (74.2)	67/97 (69.1)	65/97 (66.7)	58/97 (59.6)	67/97 (69.1)	46/97 (47.4)	46/97 (47.4)

AO, as observed; APS, abdominal pain score; CDAI, Crohn's Disease Activity Index; mNRI, modified nonresponder imputation; NRI, nonresponder imputation; SF, stool frequency.
CDAI clinical remission was defined as CDAI <150.
SF/APS clinical remission was defined as average daily SF ≤2.8 and not worse than baseline of the induction study, and average daily APS ≤1 and not worse than baseline of the induction study.

Supplementary Table 3. Clinical remission among patients who received rescue therapy

Endpoint	Week[a]	*n*/*N*	Responder Rate (95% CI)
CDAI remission	Week 0	2/16	12.5 (0.0, 28.7)
	Week 24	9/14	64.3 (39.2, 89.4)
	Week 48	5/5	100.0 (100.0, 100.0)
SF/APS remission	Week 0	0/16	0
	Week 24	8/14	57.1 (31.2, 83.1)
	Week 48	5/5	100.0 (100.0, 100.0)

AO, as observed; APS, abdominal pain score; CDAI, Crohn's Disease Activity Index SF, stool frequency.
Patients could receive ≤2 rescue therapy visits per year ≥16 weeks apart.
CDAI clinical remission was defined as CDAI <150.
SF/APS clinical remission was defined as average daily SF ≤2.8 and not worse than baseline of the induction study, and average daily APS ≤1 and not worse than baseline of the induction study.
[a]Week 24 and week 48 results are reported for the AO analysis performed with the nominal visits, which were recalibrated every 24 weeks from the date of the initial rescue therapy; week 0 results reflect the first scheduled visit of part 2.

Supplementary Table 4. Change from baseline of part 1 in hs-CRP and FCP

Biomarker	Week	*n*	Visit mean	Visit median	Change from baseline[a]	
					Mean (SD)	Median (range)
hs-CRP, mg/L	Baseline[b]	215	18.9	7.9		
	Week 52	215	6.2	3.1	−12.7 (25.8)	−3.4 (−165.0, 29.2)
	Week 76	205	5.1	2.7	−13.8 (24.9)	−4.2 (−162.3, 16.4)
	Week 100	186	4.4	2.9	−15.2 (26.7)	−4.4 (−152.1, 23.7)
FCP, µg/g	Baseline[b]	183	2278	1040		
	Week 52	183	559	147	−1719 (3735)	−538 (−24518, 4255)
	Week 76	150	552	144	−1559 (3301)	−701 (−24512, 7494)
	Week 100	139	403	134	−1971 (3843)	−779 (−24944, 1577)

hs-CRP, high-sensitivity C-reactive protein; FCP, fecal calprotectin.
[a]Based on patients with data at baseline of part 1 and the corresponding visit.
[b]Based on patients with data at baseline of part 1 and week 52 (beginning of part 2).

SUPPLEMENTARY FIGURE

Supplementary Figure 1. Sustained clinical remission at week 100. Error bars are 95% CI. Values in parentheses are *n*/*N*. Sustained clinical remission was defined as achievement of clinical remission in patients who had achieved clinical remission at the beginning of part 2. CDAI clinical remission was defined as CDAI <150. SF/APS clinical remission was defined as average daily SF ≤2.8 and not worse than baseline of the induction study and average daily APS ≤1 and not worse than baseline of the induction study. AO, as observed; APS, abdominal pain score; CDAI, Crohn's Disease Activity Index; mNRI, modified nonresponder imputation; NRI, nonresponder imputation; SF, stool frequency.

Alt text: Bar graph depicting sustained clinical remission at week 100.

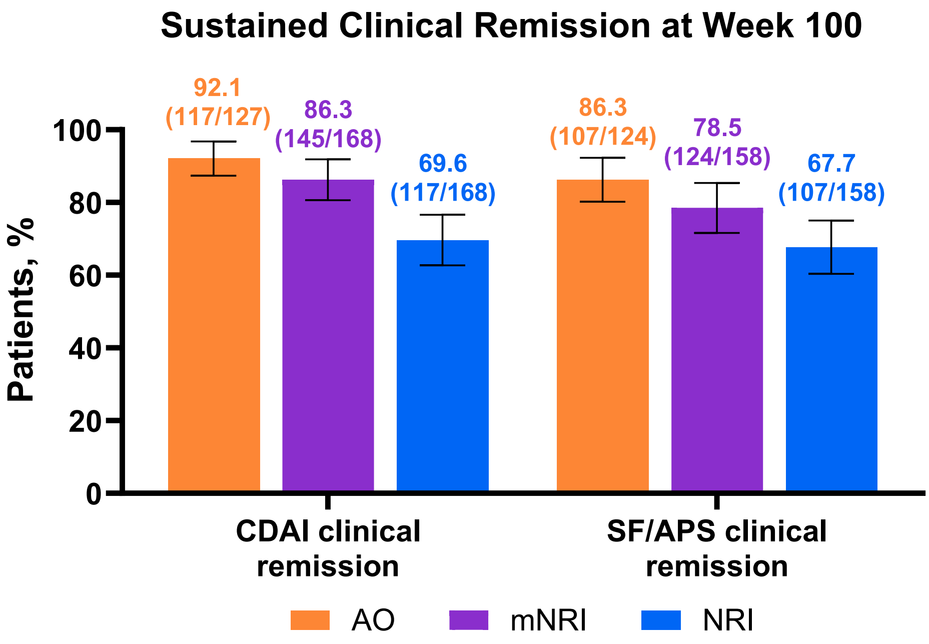

Supplement: jjaf213_Supplementary_Data [file jjaf213_supplementary_data.pdf]
